# Supplementary material for: A robust ex vivo experimental platform for molecular-genetic dissection of adult human neocortical cell types and circuits
Source: Sci Rep. 2018 May 30;8:8407. doi: 10.1038/s41598-018-26803-9 (PMC5976666; doi:10.1038/s41598-018-26803-9)
Supplement: Supplementary file 1 — Supplementary Data [file 41598_2018_26803_MOESM1_ESM.pdf]

Supplementary Material

A robust *ex vivo* experimental platform for molecular-genetic dissection of adult human neocortical cell types and circuits

Jonathan T. Ting[1], Brian Kalmbach[1,2], Peter Chong[1], Rebecca de Frates[1], C. Dirk Keene[3], Ryder P. Gwinn[4], Charles Cobbs[5], Andrew L. Ko[6,7], Jeffrey G. Ojemann[6,7], Richard G. Ellenbogen[7], Christof Koch[1], and Ed Lein[1,7]

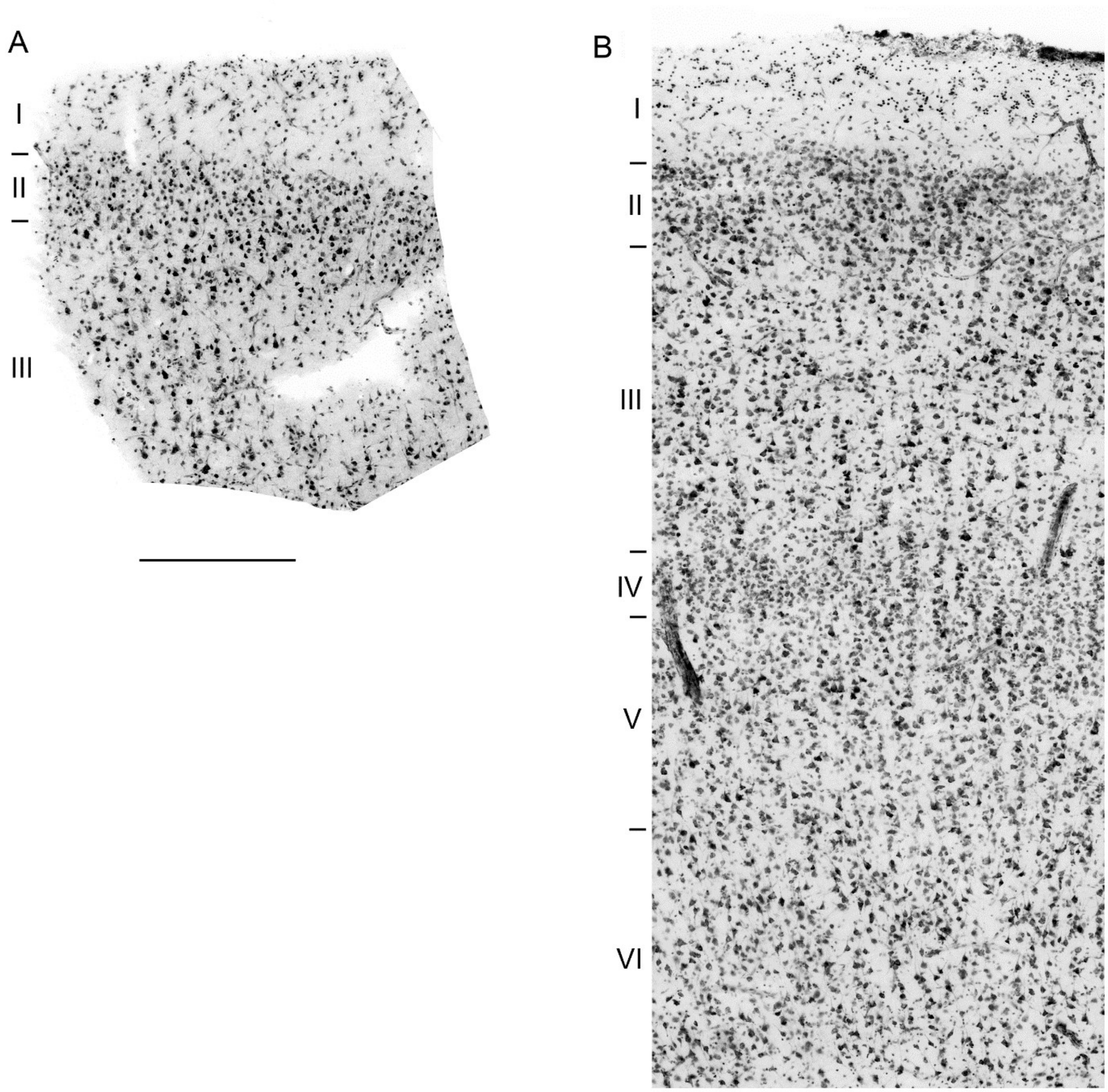

Supplementary Figure S1: Laminar architecture is well-maintained, with no overt neuron loss after 72 hours of tissue incubation post-slicing. (A) Nissl stain of a human neocortical brain slice fixed at 5 hr post-slicing. (B) Nissl stain on a human neocortical brain slice fixed at 72 hr post-slicing. Section thickness is 25 µm. Layer boundaries are indicated. Scale bar (applies to panels A and B): 300 µm. Note that the 5 hr slice dimensions were much smaller relative to the 72 hr slice from the outset; however, layers 2 and 3 are represented. All of the patch clamp recordings included in Figure 1 were carried out from these specific layers.

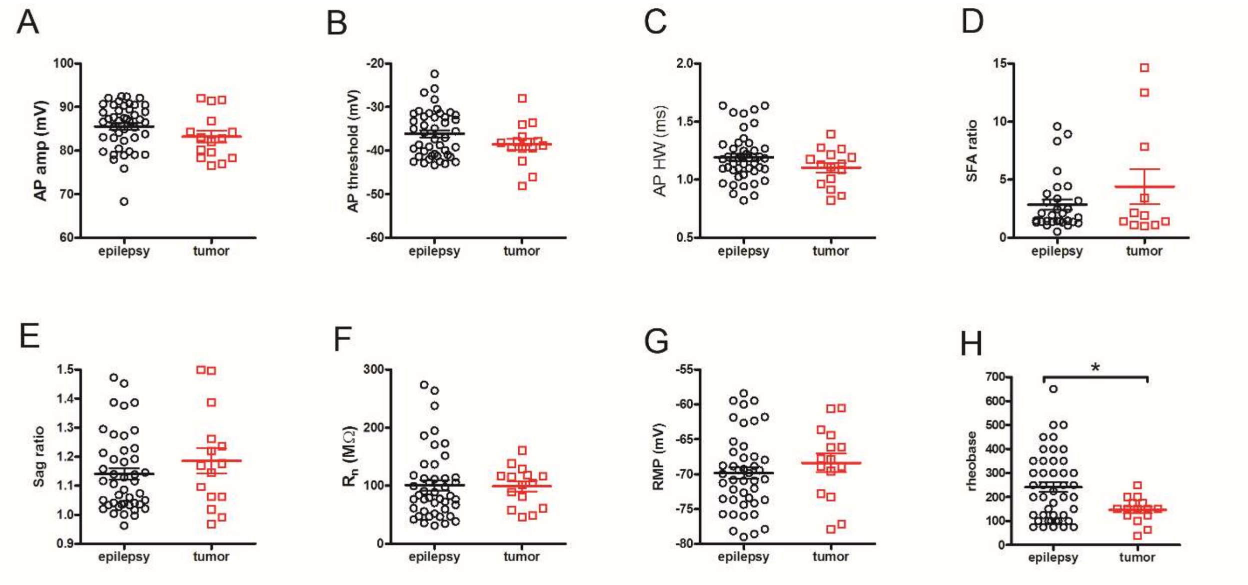

Supplementary Figure S2: Comparison of Intrinsic membrane properties of neocortical pyramidal neurons in human *ex vivo* brain slices for tumor versus epilepsy cases. (A-H) Plots of various electrophysiological features measured from 0-12 hours post-slicing for epilepsy versus tumor cases. Rn, input resistance; RMP, resting membrane potential; SFA, spike frequency adaptation; AP, action potential. Red squares correspond to neurons from tumor cases (n=11-15); black circles correspond to neurons from epilepsy cases (n=30-44). * $p < 0.05$, Mann-Whitney U test.

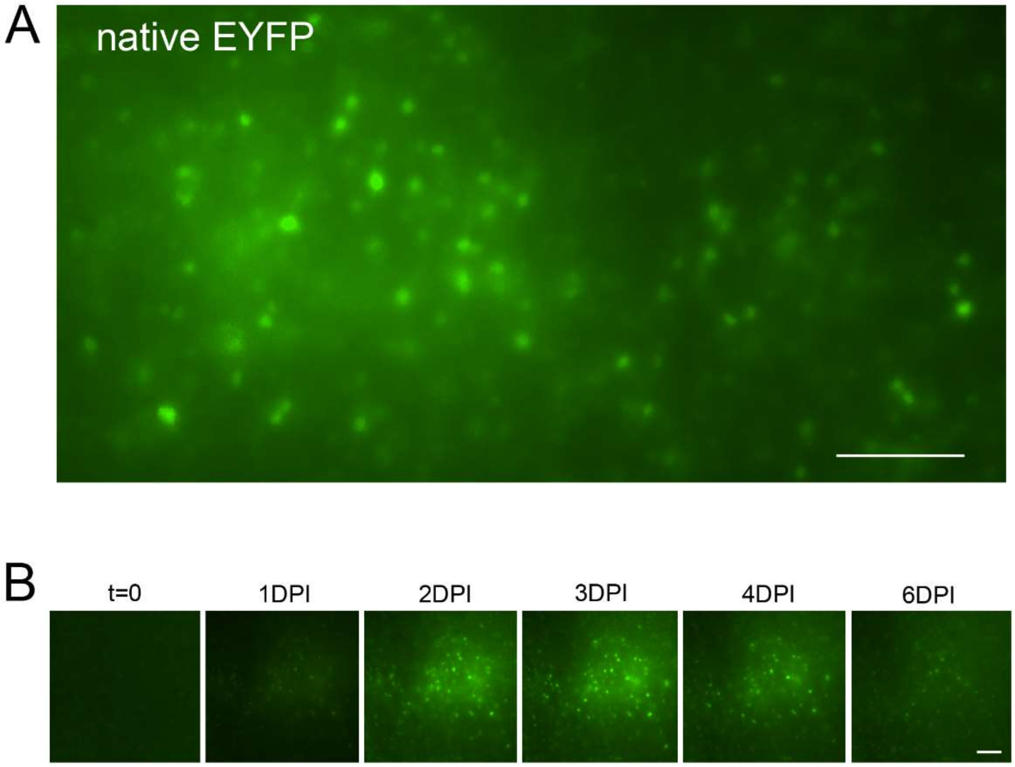

Supplementary Figure S3: Time course of HSV-mediated transgene expression in human *ex vivo* brain slice cultures. Live tissue native EYFP epifluorescence imaging of a 300 µm-thick human neocortical brain slice in culture at 2 DIV/DPI following viral infection with HSV-CaMKIIα-EYFP. Time course analysis with images collected using matched excitation exposure time (700 ms) to demonstrate the rapid onset and peak transgene expression time of 2-3 DPI following HSV transduction. Scale bars in A and B: 100 µm.

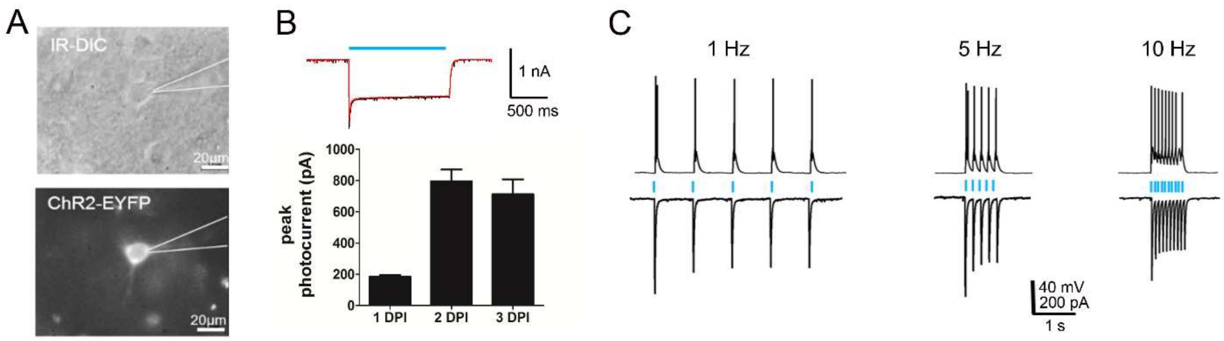

Supplementary Figure S4: Functional manipulation of neuron firing with blue light following viral expression of ChR2-EYFP. (A) IR-DIC image (top) and epifluorescence image (bottom) of a human neocortical pyramidal neuron infected with HSV-ChR2-EYFP. (B) Representative trace (top) and summary plot (bottom) of peak photocurrent amplitude evoked by blue light at 1, 2, and 3 days post virus infection (n=3-6 neurons). (C) Blue light-evoked action potential firing (top) and photocurrents (bottom) recorded from the same neuron at 1, 5, and 10 Hz stimulation frequency. These data were derived from a single surgical specimen.

Supplementary Movie S1: Spontaneous and carbachol-evoked Ca^{2+}-transients in human hippocampal dentate gyrus granule cells infected with HSV-GCaMP6s, related to analysis shown in Figure 6b. Playback is 3x faster than real time. Baseline spontaneous activity and the timing of two successive bouts of carbachol-evoked activity are indicated with subtitles. Imaging was performed at 4 DIV/DPI.
